# Supplementary material for: Entropy-Based Financial Asset Pricing
Source: PLoS One. 2014 Dec 29;9(12):e115742. doi: 10.1371/journal.pone.0115742 (PMC4278763; doi:10.1371/journal.pone.0115742)
Supplement: S1 Table — Descriptive statistics. (DOCX) [file pone.0115742.s001.docx]

Table S1. **Descriptive statistics**

| **Company** | **CRSP Ticker** | $\overline{r-r_{f}}$ | **Kurtosis** | **Skewness** | **J-B test** | **conf. level** | $\sigma$ | $\beta$ | $H_{1}$ | $H_{2}$ |
| --- | --- | --- | --- | --- | --- | --- | --- | --- | --- | --- |
| Honeywell International Inc | 10145 | 0.0345 | 23.23 | 0.24 | 152936.9 | *** | 2.08 | 1.09 | 7.47 | 5.82 |
| Beam Inc | 10225 | 0.0274 | 7.35 | 0.34 | 15423.5 | *** | 1.74 | 0.83 | 6.33 | 4.85 |
| Archer Daniels Midland Co | 10516 | 0.0409 | 8.20 | -0.03 | 19040.3 | *** | 2.04 | 0.85 | 7.19 | 5.92 |
| Brown Shoe Co Inc New | 10866 | 0.0173 | 12.33 | 0.36 | 43187.4 | *** | 2.75 | 1.13 | 9.34 | 6.78 |
| Brunswick Corp | 10874 | 0.0397 | 27.25 | 0.67 | 210865.6 | *** | 2.98 | 1.46 | 10.12 | 7.67 |
| Unisys Corp | 10890 | 0.0240 | 34.01 | 1.36 | 329786.2 | *** | 3.85 | 1.43 | 11.95 | 9.09 |
| Coca Cola Co | 11308 | 0.0457 | 15.97 | -0.05 | 72219.0 | *** | 1.60 | 0.72 | 5.96 | 4.73 |
| Consolidated Edison Inc | 11404 | 0.0120 | 9.01 | -0.26 | 23055.0 | *** | 1.21 | 0.47 | 4.55 | 3.70 |
| D T E Energy Co | 11674 | 0.0109 | 6.41 | 0.14 | 11653.2 | *** | 1.33 | 0.54 | 4.66 | 3.93 |
| Du Pont E I De Nemours & Co | 11703 | 0.0256 | 5.09 | -0.11 | 7350.3 | *** | 1.80 | 1.00 | 6.88 | 5.42 |
| Eaton Corp | 11762 | 0.0358 | 14.17 | -0.07 | 56849.8 | *** | 1.79 | 0.97 | 6.61 | 5.11 |
| Exxon Mobil Corp | 11850 | 0.0365 | 16.78 | -0.03 | 79771.0 | *** | 1.58 | 0.81 | 5.88 | 4.74 |
| General Dynamics Corp | 12052 | 0.0291 | 10.59 | 0.06 | 31780.3 | *** | 1.74 | 0.67 | 6.30 | 4.81 |
| General Electric Co | 12060 | 0.0310 | 8.85 | 0.15 | 22210.9 | *** | 1.85 | 1.19 | 6.74 | 5.21 |
| Goodrich Corp | 12140 | 0.0386 | 10.02 | -0.18 | 28476.5 | *** | 2.06 | 1.00 | 7.41 | 5.66 |
| International Business Machs Cor | 12490 | 0.0268 | 10.53 | -0.03 | 31426.9 | *** | 1.81 | 0.94 | 6.63 | 5.12 |
| I T T Corp | 12570 | 0.0424 | 8.30 | 0.15 | 19520.1 | *** | 1.74 | 0.91 | 6.54 | 5.11 |
| N L Industries Inc | 13303 | 0.0539 | 6.45 | 0.48 | 12029.3 | *** | 3.19 | 1.09 | 10.96 | 8.49 |
| P G & E Corp | 13688 | 0.0165 | 64.14 | -0.44 | 1165866.4 | *** | 1.92 | 0.57 | 5.83 | 4.70 |
| Pepsico Inc | 13856 | 0.0479 | 6.39 | 0.30 | 11679.8 | *** | 1.66 | 0.67 | 6.18 | 4.86 |
| Conocophillips | 13928 | 0.0384 | 6.81 | 0.00 | 13119.8 | *** | 1.96 | 0.85 | 7.28 | 5.91 |
| Schlumberger Ltd | 14277 | 0.0403 | 5.56 | -0.11 | 8771.8 | *** | 2.26 | 1.07 | 8.68 | 6.87 |
| Chevron Corp New | 14541 | 0.0360 | 10.18 | 0.09 | 29339.8 | *** | 1.64 | 0.80 | 6.23 | 5.02 |
| Apple Inc | 14593 | 0.1006 | 19.34 | -0.40 | 106200.6 | *** | 3.04 | 1.25 | 11.30 | 8.91 |
| Sunoco Inc | 14656 | 0.0249 | 11.30 | -0.07 | 36196.2 | *** | 2.14 | 0.95 | 7.83 | 6.19 |
| Timken Company | 14795 | 0.0262 | 11.36 | 0.05 | 36567.0 | *** | 2.28 | 1.12 | 8.13 | 6.20 |
| Foot Locker Inc | 15456 | 0.0327 | 7.92 | 0.37 | 17928.8 | *** | 2.64 | 1.02 | 9.45 | 7.19 |
| Radioshack Corp | 15560 | 0.0258 | 9.35 | -0.03 | 24747.5 | *** | 2.62 | 1.10 | 9.58 | 7.38 |
| Texas Instruments Inc | 15579 | 0.0584 | 4.93 | 0.18 | 6916.6 | *** | 2.76 | 1.36 | 10.58 | 8.31 |
| Goodyear Tire & Rubber Co | 16432 | 0.0232 | 7.68 | -0.10 | 16738.5 | *** | 2.74 | 1.35 | 9.83 | 7.32 |
| Hershey Co | 16600 | 0.0410 | 20.21 | 0.39 | 115873.6 | *** | 1.64 | 0.60 | 5.99 | 4.72 |
| Kroger Company | 16678 | 0.0455 | 140.83 | -4.09 | 5638557.9 | *** | 2.23 | 0.70 | 7.68 | 6.45 |
| C V S Caremark Corp | 17005 | 0.0372 | 11.57 | -0.34 | 38022.0 | *** | 1.94 | 0.76 | 7.11 | 5.51 |
| Bassett Furniture Industries Inc | 17137 | 0.0103 | 17.47 | 0.71 | 87018.0 | *** | 2.82 | 0.60 | 9.35 | 7.08 |
| General Mills Inc | 17144 | 0.0357 | 6.06 | 0.28 | 10502.3 | *** | 1.36 | 0.49 | 5.13 | 4.04 |
| Crown Holdings Inc | 17726 | 0.0539 | 35.18 | 1.68 | 353824.8 | *** | 2.92 | 0.93 | 8.80 | 6.51 |
| Kimberly Clark Corp | 17750 | 0.0338 | 15.79 | -0.49 | 70949.8 | *** | 1.55 | 0.61 | 5.71 | 4.44 |
| United Technologies Corp | 17830 | 0.0406 | 13.94 | -0.54 | 55357.7 | *** | 1.76 | 0.95 | 6.65 | 5.28 |
| Briggs & Stratton Corp | 17961 | 0.0158 | 5.13 | 0.10 | 7469.0 | *** | 1.98 | 0.84 | 7.18 | 5.49 |
| Procter & Gamble Co | 18163 | 0.0398 | 46.91 | -1.60 | 626337.4 | *** | 1.57 | 0.67 | 5.63 | 4.42 |
| Penney J C Co Inc | 18403 | 0.0279 | 5.56 | 0.38 | 8916.7 | *** | 2.35 | 1.11 | 8.70 | 6.55 |
| Caterpillar Inc | 18542 | 0.0521 | 5.90 | -0.10 | 9871.9 | *** | 2.08 | 1.09 | 7.86 | 6.19 |
| Colgate Palmolive Co | 18729 | 0.0470 | 12.73 | 0.20 | 45929.3 | *** | 1.62 | 0.66 | 6.00 | 4.71 |
| F M C Corp | 19166 | 0.0553 | 15.84 | -0.22 | 71105.3 | *** | 2.07 | 1.05 | 7.18 | 5.43 |
| Deere & Co | 19350 | 0.0481 | 4.59 | 0.02 | 5957.7 | *** | 2.17 | 1.05 | 8.30 | 6.51 |
| Bristol Myers Squibb Co | 19393 | 0.0257 | 14.28 | -0.45 | 58029.4 | *** | 1.79 | 0.82 | 6.57 | 5.11 |
| Walgreen Co | 19502 | 0.0472 | 5.05 | 0.08 | 7228.8 | *** | 1.83 | 0.80 | 6.92 | 5.53 |
| Boeing Co | 19561 | 0.0349 | 5.87 | -0.04 | 9747.1 | *** | 1.94 | 0.94 | 7.37 | 5.86 |
| Abbott Laboratories | 20482 | 0.0443 | 4.60 | -0.16 | 6013.1 | *** | 1.68 | 0.69 | 6.48 | 5.19 |
| Genesco Inc | 21055 | 0.0865 | 9.93 | -0.03 | 27957.9 | *** | 3.61 | 1.16 | 11.95 | 9.92 |
| Lockheed Martin Corp | 21178 | 0.0273 | 12.85 | 0.04 | 46785.2 | *** | 1.83 | 0.63 | 6.68 | 5.13 |
| Meadwestvaco Corp | 21186 | 0.0193 | 10.04 | -0.54 | 28871.5 | *** | 2.04 | 1.08 | 7.51 | 5.90 |
| International Paper Co | 21573 | 0.0197 | 11.32 | 0.08 | 36283.5 | *** | 2.19 | 1.13 | 7.92 | 6.13 |
| Exelon Corp | 21776 | 0.0226 | 8.63 | 0.15 | 21126.5 | *** | 1.58 | 0.60 | 5.47 | 4.61 |
| Pfizer Inc | 21936 | 0.0376 | 4.25 | -0.13 | 5143.2 | *** | 1.82 | 0.86 | 7.02 | 5.57 |
| Cooper Industries Plc | 21979 | 0.0338 | 15.95 | -0.16 | 72119.9 | *** | 1.98 | 1.03 | 7.19 | 5.58 |
| Corning Inc | 22293 | 0.0525 | 11.84 | -0.18 | 39784.4 | *** | 2.94 | 1.36 | 10.09 | 7.44 |
| P P G Industries Inc | 22509 | 0.0346 | 7.18 | 0.03 | 14598.0 | *** | 1.81 | 1.00 | 6.81 | 5.31 |
| Merck & Co Inc New | 22752 | 0.0400 | 12.81 | -0.56 | 46875.5 | *** | 1.77 | 0.80 | 6.65 | 5.27 |
| Motorola Solutions Inc | 22779 | 0.0427 | 7.02 | -0.13 | 13965.6 | *** | 2.69 | 1.34 | 9.99 | 7.71 |
| Firstenergy Corp | 23026 | 0.0123 | 12.25 | 0.19 | 42562.6 | *** | 1.47 | 0.58 | 4.72 | 4.19 |
| Heinz H J Co | 23077 | 0.0263 | 4.27 | 0.17 | 5186.7 | *** | 1.48 | 0.57 | 5.51 | 4.43 |
| Textron Inc | 23579 | 0.0354 | 43.20 | 0.28 | 528897.2 | *** | 2.42 | 1.26 | 7.96 | 6.02 |
| Public Service Enterprise Gp Inc | 23712 | 0.0147 | 10.72 | 0.12 | 32556.5 | *** | 1.49 | 0.64 | 5.12 | 4.26 |
| Halliburton Company | 23819 | 0.0451 | 14.74 | -0.32 | 61698.0 | *** | 2.71 | 1.16 | 10.09 | 7.95 |
| X C E L Energy Inc | 23931 | 0.0112 | 80.19 | -1.88 | 1826075.6 | *** | 1.57 | 0.56 | 4.97 | 3.87 |
| Entergy Corp New | 24010 | 0.0219 | 14.53 | -0.06 | 59775.6 | *** | 1.62 | 0.53 | 5.53 | 4.46 |
| Nextera Energy Inc | 24205 | 0.0182 | 14.72 | 0.00 | 61414.6 | *** | 1.35 | 0.55 | 4.70 | 3.80 |
| Pitney Bowes Inc | 24459 | 0.0220 | 20.00 | -1.02 | 114456.4 | *** | 1.81 | 0.88 | 6.66 | 5.31 |
| Alcoa Inc | 24643 | 0.0216 | 9.67 | 0.10 | 26513.2 | *** | 2.38 | 1.25 | 8.68 | 6.73 |
| Raytheon Co | 24942 | 0.0243 | 65.90 | -1.87 | 1234405.7 | *** | 1.81 | 0.60 | 6.24 | 5.02 |
| Oneok Inc New | 25232 | 0.0398 | 25.03 | -0.06 | 177473.5 | *** | 1.95 | 0.79 | 6.68 | 5.44 |
| Campbell Soup Co | 25320 | 0.0285 | 6.92 | 0.38 | 13722.7 | *** | 1.67 | 0.59 | 6.15 | 4.73 |
| Whirlpool Corp | 25419 | 0.0195 | 6.17 | 0.17 | 10832.3 | *** | 2.23 | 1.06 | 8.29 | 6.34 |
| Harris Corp | 25582 | 0.0346 | 8.18 | 0.13 | 18983.7 | *** | 2.15 | 0.98 | 7.90 | 6.16 |
| Ford Motor Co Del | 25785 | 0.0352 | 14.61 | 0.55 | 60820.6 | *** | 2.50 | 1.16 | 8.97 | 7.00 |
| Disney Walt Co | 26403 | 0.0549 | 13.12 | -0.13 | 48767.4 | *** | 2.02 | 1.13 | 7.49 | 5.88 |
| Biglari Holdings Inc | 26607 | 0.0854 | 57.58 | -0.55 | 939711.1 | *** | 3.43 | 0.74 | 11.54 | 9.22 |
| A S A Gold & Precious Metals Ltd | 26649 | 0.0150 | 5.32 | 0.25 | 8081.3 | *** | 2.18 | 0.15 | 8.11 | 6.27 |
| Kellogg Co | 26825 | 0.0311 | 20.76 | 0.09 | 122114.8 | *** | 1.61 | 0.62 | 5.88 | 4.62 |
| Ryder Systems Inc | 27633 | 0.0267 | 5.05 | -0.13 | 7253.2 | *** | 2.15 | 1.06 | 7.89 | 6.33 |
| Hewlett Packard Co | 27828 | 0.0439 | 6.64 | 0.01 | 12476.1 | *** | 2.45 | 1.22 | 9.23 | 7.19 |
| Baxter International Inc | 27887 | 0.0349 | 15.33 | -0.94 | 67607.8 | *** | 1.87 | 0.72 | 6.86 | 5.56 |
| Duke Energy Corp New | 27959 | 0.0191 | 13.89 | -0.12 | 54692.0 | *** | 1.45 | 0.55 | 5.11 | 3.97 |
| Xerox Corp | 27983 | 0.0202 | 21.10 | 0.29 | 126269.3 | *** | 2.56 | 1.11 | 8.55 | 6.45 |
| Nacco Industries Inc | 28118 | 0.0451 | 7.60 | 0.38 | 16528.6 | *** | 2.68 | 1.18 | 9.44 | 6.98 |
| Unilever N V | 28310 | 0.0429 | 69.75 | 0.15 | 1378374.3 | *** | 1.67 | 0.75 | 5.83 | 4.61 |
| Hess Corp | 28484 | 0.0371 | 9.72 | -0.48 | 27037.4 | *** | 2.20 | 0.98 | 8.08 | 6.28 |
| Masco Corp | 34032 | 0.0171 | 5.88 | 0.08 | 9814.6 | *** | 2.32 | 1.14 | 8.28 | 6.34 |
| Occidental Petroleum Corp | 34833 | 0.0329 | 9.00 | -0.02 | 22927.0 | *** | 2.04 | 0.93 | 7.37 | 5.95 |
| Sherwin Williams Co | 36468 | 0.0495 | 9.48 | -0.09 | 25478.9 | *** | 1.89 | 0.84 | 6.95 | 5.47 |
| Williams Cos | 38156 | 0.0633 | 124.54 | 2.06 | 4399507.1 | *** | 3.15 | 1.21 | 9.50 | 7.35 |
| Donnelley R R & Sons Co | 38682 | 0.0043 | 16.19 | -0.33 | 74341.2 | *** | 1.88 | 0.97 | 6.84 | 5.36 |
| Wells Fargo & Co New | 38703 | 0.0609 | 27.36 | 1.45 | 214537.1 | *** | 2.36 | 1.24 | 7.73 | 5.80 |
| Skyline Corp | 38850 | -0.0059 | 7.96 | 0.37 | 18127.9 | *** | 2.41 | 0.91 | 8.24 | 6.29 |
| Mattel Inc | 39538 | 0.0473 | 11.74 | -0.11 | 39033.4 | *** | 2.42 | 0.88 | 8.70 | 6.92 |
| Becton Dickinson & Co | 39642 | 0.0487 | 11.53 | -0.28 | 37757.7 | *** | 1.70 | 0.64 | 6.31 | 4.89 |
| Computer Sciences Corp | 40125 | 0.0438 | 21.16 | -0.96 | 127892.3 | *** | 2.24 | 1.02 | 8.02 | 6.18 |
| Avon Products Inc | 40416 | 0.0341 | 20.60 | -0.51 | 120504.3 | *** | 2.12 | 0.80 | 7.34 | 5.56 |
| Cummins Inc | 41080 | 0.0456 | 7.69 | 0.26 | 16844.8 | *** | 2.42 | 1.20 | 8.72 | 6.53 |
| Con Way Inc | 41929 | 0.0252 | 4.77 | 0.13 | 6466.2 | *** | 2.56 | 1.05 | 9.23 | 7.11 |
| Officemax Inc New | 42024 | 0.0001 | 20.60 | 0.94 | 121181.1 | *** | 2.86 | 1.25 | 9.59 | 7.20 |
| Meredith Corp | 42796 | 0.0246 | 7.01 | 0.24 | 13988.2 | *** | 1.87 | 0.92 | 6.76 | 5.13 |
| Allegheny Technologies | 43123 | 0.0370 | 6.97 | 0.40 | 13924.5 | *** | 2.85 | 1.33 | 10.09 | 7.49 |
| Stanley Black & Decker Inc | 43350 | 0.0340 | 5.14 | 0.23 | 7538.8 | *** | 1.99 | 1.01 | 7.27 | 5.74 |
| Mcdonalds Corp | 43449 | 0.0500 | 5.37 | -0.08 | 8161.7 | *** | 1.65 | 0.70 | 6.31 | 5.09 |
| V F Corp | 43553 | 0.0466 | 9.88 | -0.07 | 27649.0 | *** | 1.95 | 0.88 | 7.17 | 5.47 |
| Bemis Co Inc | 43772 | 0.0434 | 4.72 | -0.07 | 6304.4 | *** | 1.82 | 0.85 | 6.79 | 5.46 |
| Automatic Data Processing Inc | 44644 | 0.0458 | 16.10 | -0.53 | 73763.2 | *** | 1.70 | 0.88 | 6.33 | 4.92 |
| Supervalu Inc | 44951 | 0.0061 | 8.85 | -0.58 | 22556.5 | *** | 2.07 | 0.72 | 7.32 | 5.78 |
| Rowan Companies Inc | 45495 | 0.0526 | 2.87 | 0.22 | 2394.8 | *** | 3.19 | 1.23 | 11.34 | 9.59 |
| Clorox Co | 46578 | 0.0401 | 10.59 | -0.24 | 31813.0 | *** | 1.62 | 0.59 | 5.86 | 4.51 |
| Genuine Parts Co | 46674 | 0.0225 | 5.09 | 0.20 | 7377.3 | *** | 1.47 | 0.73 | 5.29 | 4.06 |
| Bard C R Inc | 46877 | 0.0527 | 7.54 | 0.08 | 16098.4 | *** | 1.92 | 0.70 | 7.09 | 5.50 |
| Rite Aid Corp | 46922 | 0.0228 | 22.02 | 0.70 | 137871.8 | *** | 3.54 | 1.11 | 10.73 | 7.83 |
| New York Times Co | 47466 | 0.0059 | 8.95 | 0.60 | 23087.3 | *** | 2.24 | 0.98 | 7.96 | 6.08 |
| C N A Financial Corp | 47626 | 0.0220 | 24.01 | -0.01 | 163290.3 | *** | 2.13 | 1.10 | 7.05 | 5.29 |
| Jpmorgan Chase & Co | 47896 | 0.0312 | 12.89 | 0.46 | 47271.5 | *** | 2.51 | 1.49 | 8.79 | 6.62 |
| Gannett Inc | 47941 | 0.0102 | 30.17 | 0.79 | 258519.3 | *** | 2.20 | 1.10 | 7.41 | 5.68 |
| Moodys Corp | 48506 | 0.0352 | 8.44 | 0.01 | 20196.4 | *** | 2.02 | 1.00 | 7.04 | 5.25 |
| Union Pacific Corp | 48725 | 0.0396 | 6.61 | -0.23 | 12440.7 | *** | 1.76 | 0.89 | 6.71 | 5.25 |
| Lincoln National Corp In | 49015 | 0.0357 | 48.23 | 0.95 | 659983.7 | *** | 2.88 | 1.48 | 8.08 | 6.13 |
| Target Corp | 49154 | 0.0507 | 13.01 | -0.30 | 48075.4 | *** | 2.14 | 1.10 | 7.99 | 6.18 |
| Potlatch Corp New | 49744 | 0.0212 | 14.43 | 0.13 | 58970.7 | *** | 2.17 | 1.15 | 7.67 | 5.93 |
| Lilly Eli & Co | 50876 | 0.0350 | 18.57 | -0.65 | 98182.3 | *** | 1.83 | 0.81 | 6.85 | 5.43 |
| Tenet Healthcare Corp | 52337 | 0.0220 | 48.65 | 0.43 | 670845.7 | *** | 2.92 | 0.87 | 9.68 | 7.58 |
| Grainger W W Inc | 52695 | 0.0468 | 6.05 | 0.23 | 10416.0 | *** | 1.72 | 0.83 | 6.48 | 5.04 |
| Hasbro Inc | 52978 | 0.0421 | 15.23 | 0.08 | 65730.8 | *** | 2.20 | 0.85 | 8.00 | 6.29 |
| S P X Corp | 55212 | 0.0384 | 12.66 | -0.71 | 45995.5 | *** | 2.50 | 1.07 | 8.75 | 6.76 |
| Wal Mart Stores Inc | 55976 | 0.0582 | 3.53 | 0.15 | 3553.1 | *** | 1.82 | 0.87 | 6.83 | 5.46 |
| Louisiana Pacific Corp | 56223 | 0.0314 | 9.68 | 0.07 | 26554.8 | *** | 2.97 | 1.39 | 10.40 | 7.91 |
| Conagra Inc | 56274 | 0.0299 | 12.82 | -0.55 | 46934.9 | *** | 1.67 | 0.60 | 6.11 | 4.73 |
| Ball Corp | 57568 | 0.0405 | 8.42 | 0.05 | 20086.1 | *** | 1.87 | 0.84 | 6.87 | 5.42 |
| American Express Co | 59176 | 0.0452 | 9.53 | 0.13 | 25726.5 | *** | 2.35 | 1.45 | 8.56 | 6.53 |
| Chubb Corp | 59192 | 0.0396 | 7.80 | 0.55 | 17562.6 | *** | 1.73 | 0.90 | 6.31 | 4.78 |
| Molson Coors Brewing Co | 59248 | 0.0356 | 7.35 | -0.17 | 15322.2 | *** | 2.12 | 0.57 | 7.42 | 5.93 |
| Intel Corp | 59328 | 0.0749 | 5.71 | -0.06 | 9242.0 | *** | 2.68 | 1.41 | 10.29 | 8.20 |
| Bank Of America Corp | 59408 | 0.0235 | 27.64 | 0.79 | 217148.6 | *** | 2.70 | 1.45 | 8.28 | 6.12 |
| Snap On Inc | 60206 | 0.0231 | 7.33 | 0.07 | 15205.6 | *** | 1.86 | 0.94 | 6.71 | 5.07 |
| Paccar Inc | 60506 | 0.0540 | 4.32 | 0.11 | 5310.9 | *** | 2.29 | 1.22 | 8.29 | 6.24 |
| Fedex Corp | 60628 | 0.0396 | 4.24 | 0.21 | 5129.6 | *** | 2.11 | 0.99 | 7.99 | 6.23 |
| Advanced Micro Devices Inc | 61241 | 0.0439 | 7.80 | -0.04 | 17236.2 | *** | 3.84 | 1.61 | 14.05 | 11.00 |
| Lowes Companies Inc | 61399 | 0.0627 | 4.45 | 0.11 | 5628.7 | *** | 2.34 | 1.10 | 8.79 | 7.01 |
| Cigna Corp | 64186 | 0.0388 | 27.91 | -0.78 | 221310.8 | *** | 2.13 | 0.92 | 7.29 | 5.69 |
| Limited Brands Inc | 64282 | 0.0586 | 4.58 | 0.16 | 5961.2 | *** | 2.49 | 1.19 | 9.33 | 7.39 |
| Norfolk Southern Corp | 64311 | 0.0389 | 5.24 | 0.04 | 7783.8 | *** | 1.96 | 0.99 | 7.41 | 5.70 |
| Dominion Resources Inc Va New | 64936 | 0.0170 | 11.67 | -0.51 | 38851.3 | *** | 1.24 | 0.51 | 4.35 | 3.47 |
| Verizon Communications Inc | 65875 | 0.0226 | 12.13 | 0.56 | 42051.6 | *** | 1.67 | 0.76 | 6.20 | 4.84 |
| A T & T Inc | 66093 | 0.0229 | 11.22 | 0.11 | 35704.5 | *** | 1.72 | 0.81 | 6.38 | 5.01 |
| American International Group Inc | 66800 | 0.0279 | 92.09 | 1.66 | 2406066.2 | *** | 3.27 | 1.40 | 8.33 | 7.09 |

*Note:* This table shows the descriptive statistics of the 150 randomly selected stocks based on daily returns adjusted by risk free rate. J-B test stands for the Jarque-Bera test, conf. level shows *** for 99% confidence level (the exact value is 9.2 for the J-B test at 99%), H1 and H2 represents the Shannon and the Rényi entropy respectively. If the J-B test value is higher than the 99% confidence level the normality null hypothesis can be rejected on 99%.
