# Supplementary material for: Entropy-Based Financial Asset Pricing
Source: PLoS One. 2014 Dec 29;9(12):e115742. doi: 10.1371/journal.pone.0115742 (PMC4278763; doi:10.1371/journal.pone.0115742)
Supplement: S2 Table — The most often used Kernel functions. (DOCX) [file pone.0115742.s002.docx]

Table S2. **The most often used Kernel functions**

| **Function name** | $K\left(z\right)$ |
| --- | --- |
| Uniform | $\frac{1}{2}I_{\left\{\left\vert z\right\vert\leq1\right\}}$ |
| Triangle | $\left(1-\left\vert z\right\vert\right)I_{\left\{\left\vert z\right\vert\leq1\right\}}$ |
| Epanechnikov | $\frac{3}{4}\left(1-z^{2}\right)I_{\left\{\left\vert z\right\vert\leq1\right\}}$ |
| Triweight | $\frac{35}{32}\left(1-z^{2}\right)^{3}I_{\left\{\left\vert z\right\vert\leq1\right\}}$ |
| Gaussian | $\frac{1}{\sqrt{2\pi}}e^{-\frac{1}{2}z^{2}}$ |

*Note:* The table collects the most often applied kernel functions used for kernel density estimation. The I is the indicator function, for practical reasons (computational time) it is recommended to use one of the indicator based kernel functions.
