# Supplementary material for: Entropy-Based Financial Asset Pricing
Source: PLoS One. 2014 Dec 29;9(12):e115742. doi: 10.1371/journal.pone.0115742 (PMC4278763; doi:10.1371/journal.pone.0115742)
Supplement: S3 Table — Explanatory power of Shannon entropy by different density estimation methods. (DOCX) [file pone.0115742.s003.docx]

Table S3. **Explanatory power of Shannon entropy by different density estimation methods**

| **Density estimation** | $\hat{\eta}_{P1}$ |  |  |  |  |
| --- | --- | --- | --- | --- | --- |
| **Histogram** | 12.98% | 13.38% | 10.15% | 0.67 | 0.62 |
| **Sample Spacing (Simple)** | 15.22% | 15.31% | 9.36% | 0.66 | 0.60 |
| **Sample Spacing (Correa)** | 14.61% | 15.27% | 9.06% | 0.66 | 0.61 |
| **Kernel (Uniform)** | 13.02% | 12.57% | 9.82% | 0.68 | 0.60 |
| **Kernel (Triangle)** | 13.21% | 13.34% | 9.76% | 0.67 | 0.59 |
| **Kernel (Epanechnikov)** | 13.22% | 13.14% | 9.80% | 0.68 | 0.59 |
| **Kernel (Triweight)** | 13.21% | 13.78% | 9.67% | 0.67 | 0.59 |
| **Kernel (Gaussian)** | 13.21% | 13.34% | 9.76% | 0.67 | 0.59 |

*Note:* To find out, that which density estimation method to be used in our study, we compare the efficiency of the ordinary applied methods. We estimate and evaluate risk measures of 150 random securities using different density estimation methods to estimate Shannon entropy by daily risk premiums in different periods: (1) long term, from 1985 to the end of 2011 (*P*1); (2) 18 10-year periods shifting by one year from period (1985-1994) to period (2002-2011) and we split the 10-year-long periods into two 5-5 year periods (*P*2*i*, *P*2*o*). The number of bins for histogram and sample-spacing based estimation is 175, and the bandwidth of kernel density estimation is calculated by simplex search optimization method. $\hat{\eta}_{P1}$ shows the explanatory power of risk measures for expected risk premium on long term, stands for the average explanatory power of risk measures evaluated in the first 5 years of 10-year shorter periods in sample. shows the average predictability power of risk measures calculated by estimating risk in the first 5 years and evaluating them on the consecutive 5 years in each 10-year periods. The 5^th^ and 6^th^ columns stand for the relative standard deviation of the explanatory and predicting power based on the 18 shorter periods for the investigated risk measures. However the sample spacing performs the best in sample, the histogram based estimation offers better tradeoff in terms of the explanatory and predicting power, for that reason we choose the latter one.
