# Supplementary material for: Entropy-Based Financial Asset Pricing
Source: PLoS One. 2014 Dec 29;9(12):e115742. doi: 10.1371/journal.pone.0115742 (PMC4278763; doi:10.1371/journal.pone.0115742)
Supplement: S4 Table — Labeling periods by market trend. (DOCX) [file pone.0115742.s004.docx]

Table S4. **Labeling periods by market trend**

| **Start date** | **End date** | **Market trend** |
| --- | --- | --- |
| 1985-01-02 | 2000-01-31 | bullish |
| 2000-02-01 | 2002-08-31 | bearish |
| 2002-09-01 | 2007-04-30 | bullish |
| 2007-05-01 | 2009-01-31 | bearish |
| 2009-02-01 | 2011-04-30 | bullish |
| 2011-05-01 | 2011-08-31 | bearish |
| 2011-09-01 | 2011-12-31 | bullish |

*Note:* The table contains partition applied by explanatory power analysis by market trends. The trends are defined by the monthly returns of CRSP value weighted index. That is why the trends always start on the first trading day of the given month.
